# Think out of the box: association of left congenital diaphragmatic hernia and abnormal origin of the right pulmonary artery: A train can hide another

**DOI:** 10.1186/s12887-023-04164-1

**Published:** 2023-07-11

**Authors:** Arthur Gavotto, Pascal Amedro, Gilles Cambonie

**Affiliations:** 1 Department of Neonatal Medicine and Pediatric Intensive Care, Arnaud de Villeneuve Hospital, Montpellier University Hospital, 371 Avenue du Doyen Giraud, 34295 Montpellier, France; 2 PhyMedExp, CNRS, INSERM, University of Montpellier, Montpellier, France; 3 Paediatric and Congenital Cardiology Department, M3C National Reference Centre, Bordeaux University Hospital, Bordeaux, France; 4 IHU Liryc, INSERM 1045, Bordeaux University, Bordeaux, France; 5 Pathogenesis and Control of Chronic Infection, INSERM UMR 1058, University of Montpellier, Montpellier, France

**Keywords:** Congenital diaphragmatic hernia, Congenital heart disease, Pulmonary hypertension, Poiseuille’s law, Pediatrics

## Abstract

**Background:**

We report the occurrence of a severe pulmonary hypertension (PH) in a neonate affected by a left congenital diaphragmatic hernia (CDH). PH in this patient was associated with an abnormal origin of the right pulmonary artery from the right brachiocephalic artery. This malformation, sometimes named hemitruncus arteriosus, has to the best of our knowledge never been reported in association with a CDH.

**Case presentation:**

A male newborn was hospitalized from birth in the neonatal intensive care unit after prenatal diagnosis of a left CDH. Ultrasound examination at 34 weeks of gestational age evaluated the observed-to-expected lung-to-head ratio at 49%. Birth occurred at 38^+ 5^ weeks of gestational age. Soon after admission, severe hypoxemia, i.e., preductal pulse oximetry oxygen saturation (SpO_2_) < 80%, prompted therapeutic escalation including the use of high frequency oscillatory ventilation with fraction of inspired oxygen (FiO_2_) 100% and inhaled nitric oxide (iNO). Echocardiography assessment revealed signs of severe PH and normal right ventricle function. Despite administration of epoprostenolol, milrinone, norepinephrine, and fluid loadings with albumin and 0.9% saline, hypoxemia remained severe, preductal SpO_2_ inconsistently greater than or equal to 80-85% and post ductal SpO_2_ lower on average by 15 points. This clinical status remained unchanged during the first 7 days of life. The infant’s clinical instability was incompatible with surgical intervention, while chest X-ray showed a relatively preserved lung volume, especially on the right side. This prompted an additional echocardiography, aimed at searching an explanation of this unusual evolution and found an abnormal origin of the right pulmonary artery, which was confirmed on computed tomography angiography subsequently. A change in the medical strategy was decided, with the suspension of pulmonary vasodilator treatments, the administration of diuretics, and the decrease in norepinephrine dose to decrease the systemic-to-pulmonary shunt. Progressive improvement in the infant respiratory and hemodynamic status enabled to perform CDH surgical repair 2 weeks after birth.

**Conclusions:**

This case recalls the interest of systematic analysis of all potential causes of PH in a neonate with CDH, a condition frequently associated with various congenital malformations.

## Background

We report the occurrence of a severe pulmonary hypertension (PH) in a neonate affected by a left congenital diaphragmatic hernia (CDH) associated with an abnormal origin of the right pulmonary artery from the right brachiocephalic artery, also named right hemitruncus arteriosus.

In newborns with CDH, respiratory failure originates from abnormal lung development, which affects the alveoli as well as their vascularization. This results in pulmonary hypoplasia, but also structural and functional vascular abnormalities that cause persistent PH [1]. Underdevelopment and abnormal vasoreactivity of the pulmonary vasculature result in sustained elevation of pulmonary vascular resistance and low pulmonary blood flow, causing right-to-left shunting of blood across the ductus arteriosus and/or the foramen ovale, and, ultimately, refractory hypoxemia [2]. Management mainly consists in supporting respiratory function with mechanical ventilation, with the aim of maintaining preductal SpO_2_ > 80% and partial pressure of carbon dioxide (PCO_2_) between 50 and 70 mmHg [3]. Treatment of CDH-associated PH with vasodilator treatments such as inhaled nitric oxide (iNO), prostacyclin, sildenafil, or milrinone should be limited to severe forms after optimization of alveolar ventilation, control of stress and pain, and correction of hypovolemia [4, 5].

This standardized support may be unsuitable in very specific situations, in particular with congenital malformations. According to a recent review, 15% of neonates with CDH have congenital heart disease, of which 42% are critical [6]. Anomalous origin of the branches of the pulmonary artery to the aorta accounts for 0.12% of congenital heart defects [7]. In most cases, the right pulmonary artery arises from the posterior wall of ascending aorta, very near the aortic valve, is isolated (e.g., without intracardiac malformation), as opposed to left pulmonary artery forms [8]. From the rare cases reported in the literature, no association with genetic syndromes has been described in right hemitruncus arteriosus [9]. Without early surgical repair, the patient is exposed to severe pulmonary hypertension, and, ultimately, cardiac dysfunction [10]. This malformation has to the best of our knowledge never been reported in association with a CDH.

The challenge of this rare association (left CDH with an abnormal origin of the right pulmonary artery from the right brachiocephalic artery) was then to totally reverse the therapeutic strategy, to restore an adequate hemodynamic.

## Case presentation

A male newborn was hospitalized after birth in the neonatal intensive care unit (NICU) after prenatal diagnosis of a left congenital diaphragmatic hernia (CDH). The infant was the second child of consanguineous (first-degree cousins) and healthy North African parents. The couple had been referred at the last trimester of pregnancy to the expert center for CDH in our institution. At 34 weeks of gestational age, prenatal ultrasound showed left CDH associated with various anomalies: short corpus callosum (< 1st centile), palate cleft, azygous venous return, bilateral cryptorchidism, and intrauterine growth retardation (< 3rd centile according to the French reference values[11]). Ultrasound examination at 34 weeks of gestational age evaluated the observed-to-expected lung-to-head ratio at 49%, and magnetic resonance imaging (MRI) at 35 weeks of gestational age measured the observed-to-expected total fetal lung volume at 33%, corresponding to a mild-to-moderate severity in terms of prediction of CDH-related lung hypoplasia [12]. In addition, MRI showed a persistent portion of the anterior diaphragm, which maintained the liver in abdominal position. Standard karyotype and array comparative genomic hybridization revealed no abnormality.

Birth occurred at 38^+ 5^ weeks of gestational age by cesarean section for fetal bradycardia and membrane rupture, with a weight of 2670 g (5th centile), a height of 47 cm (8th centile), and a head circumference of 34.5 cm (55th centile). Apgar scores at 1, 5 and 10 min were, respectively, 2, 3 and 5. Arterial cord blood pH was 7.21 and cord lactate was 2.5 mmol/L. Nasotracheal intubation was achieved 2 min after birth, and the infant was transported in the NICU. Clinical examination confirmed palate cleft, left cryptorchidism with a short penis (19 mm), and distal arthrogryposis of the hands and feet. Soon after admission, severe hypoxemia, i.e., preductal pulse oximetry oxygen saturation (SpO_2_) < 80%, prompted therapeutic escalation including the use of high frequency oscillatory ventilation with fraction of inspired oxygen (FiO_2_) 100% oxygen and inhaled nitric oxide (iNO, 20 ppm). Echocardiography revealed signs of severe PH, with estimated systolic pulmonary artery pressure of 80 mmHg from peak tricuspid regurgitant jet velocity, inverted septal curvature, and a predominant right-to-left shunt in a large persistent ductus arteriosus (PDA) of 4.5 mm of diameter. In addition, echocardiography showed a normal right ventricle function (absence of dilation, tricuspid annular plane systolic excursion of 8.5 mm), and confirmed the absence of intracardiac defect, the absence of inferior vena cava, and the presence of the azygous venous return. Despite administration of epoprostenolol, milrinone, norepinephrine, and fluid loadings with albumin and 0.9% saline, hypoxemia remained severe, preductal SpO_2_ inconsistently greater than or equal to 80-85% and post ductal SpO_2_ lower on average by 15 points.

**Fig. 1:**
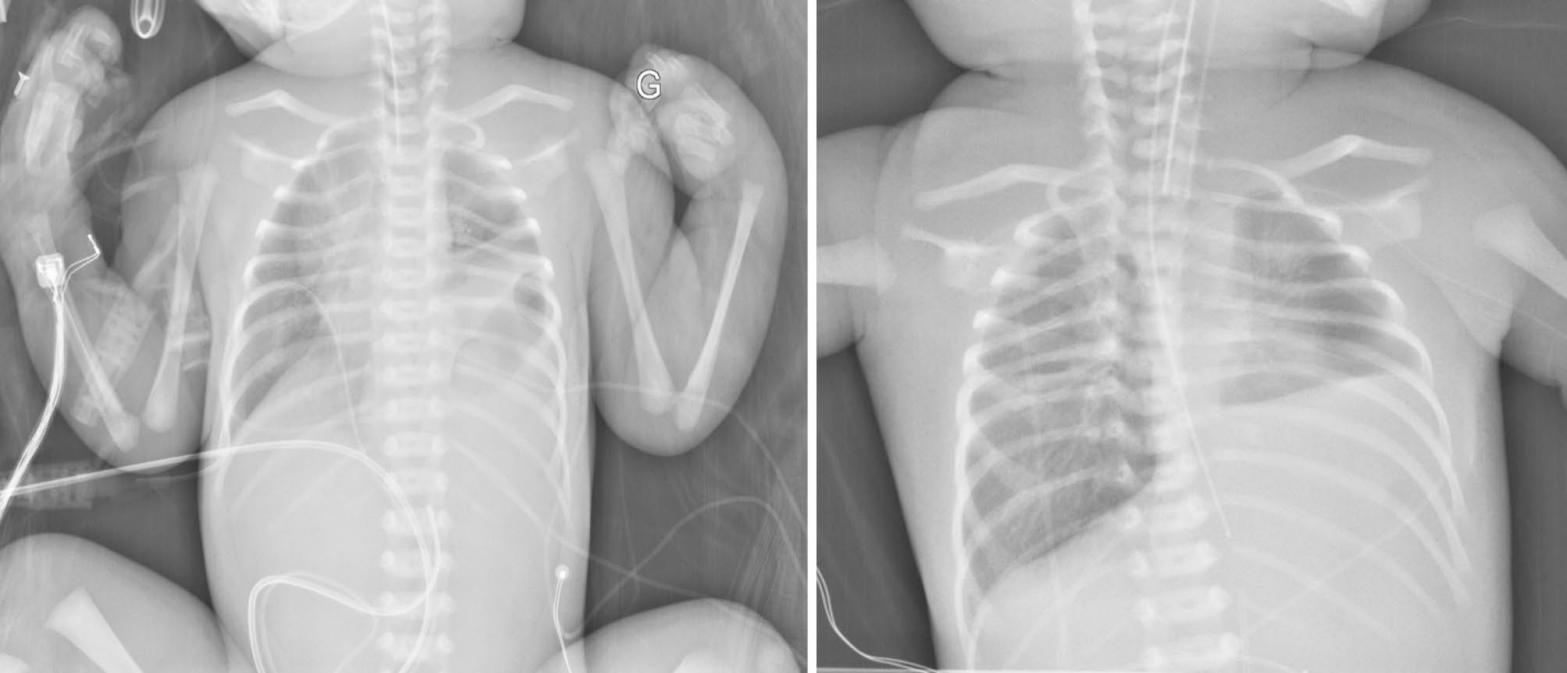
Chest X-ray at day 1 (left panel) and day 5 (right panel) of life. Chest radiography shows the bowel loops occupying part of the left hemithorax the 1st post-natal day, and a relatively preserved lung volume, especially on the right side, the 5th post-natal day

**Fig. 2:**
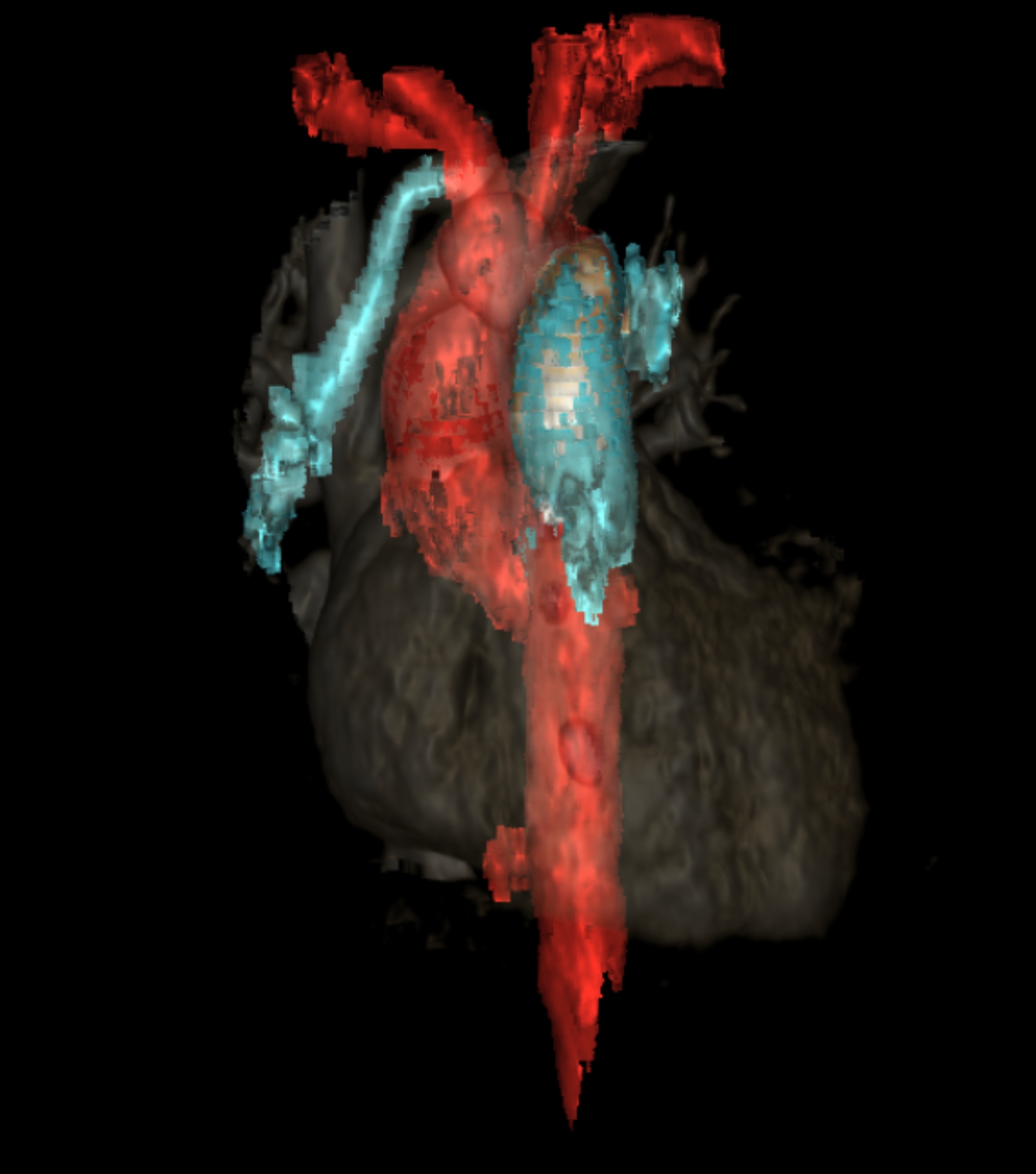
3D reconstruction of computed tomography angiography. Reconstruction showing the anomalous origin of the right pulmonary artery from the right brachiocephalic artery (indicated by the white arrow)

This clinical status remained unchanged during the first 7 days of life, with. The infant’s clinical instability was incompatible with surgical intervention, while chest X-ray showed a relatively preserved lung volume, especially on the right side (Fig. 1). This prompted an additional echocardiography, aimed at searching an explanation of this unusual evolution. The pulmonary veins were in normal position, with no stenosis, and left ventricle function was normal, with no aortic coarctation. However, the right pulmonary artery was poorly visualized, and appeared to probably originate from the right brachiocephalic artery, with significant diastolic reflux in the descending aorta and increased flow in the right pulmonary vein. This abnormal origin of the right pulmonary artery was confirmed on computed tomography angiography performed 9 days after birth (Fig. 2).

A change in the medical strategy was decided, with the suspension of pulmonary vasodilator treatments, the administration of diuretics, and the decrease in norepinephrine dose to target a minimal mean blood pressure of 35–45 mmHg to decrease the systemic-to-pulmonary shunt. Progressive improvement in the infant respiratory and hemodynamic status enabled to perform CDH surgical repair 2 weeks after birth. Then, surgical reimplantation of the right pulmonary artery in the trunk of the pulmonary artery was performed 5 weeks after birth.

## Discussion

Unstable refractory hypoxemia in an infant with CDH despite apparently appropriate treatment should question the origin of the PH and make consider multifactorial causes. Several unusual data should have drawn our attention in this observation and, first of all, uncertainty about pulmonary hypoplasia, the severity of which is often superimposed on that of PH. In this respect, there was discrepancy between lung-to-head ratio, which predicted a mild disease and total fetal lung volume, which predicted a moderate-to-severe disease [12, 13]. In addition, the satisfactory recruitment of the right lung, resulting in PCO_2_ measurements regularly below the target values, were not consistent with severe restrictive respiratory insufficiency.

Extracorporeal membrane oxygenation (ECMO) is a rescue support in neonatal CDH with cardio-respiratory failure, with persistent controversies about patient selection, timing of initiation and timing of surgical repair [2]. In our patient, the severity of PH and the settings of high frequency oscillatory ventilation were arguments for recourse to extracorporeal life support [14]. On the other hand, the multiple anomalies observed on prenatal ultrasound as on postnatal examination suggested a genetic abnormality or syndrome. After sharing this information with parents, and also taking into account the potential complications of ECMO,[15] we decided against this option.

The classical physiology of PH in CDH distinguishes three main phenotypes: (i) mild or non-existent PH with normally functional right ventricle; (ii) precapillary PH with predominant right-to-left PDA and atrial shunts, possibly associated with right-sided heart failure with or without secondary left-sided heart failure; and (iii) post-capillary PH with primary left-sided heart failure, and predominant right-to-left ductus arteriosus shunt and left-to-right atrial shunt [2]. According to Poiseuille equation, pulmonary artery pressure depends on pulmonary blood flow, right ventricular afterload and left atrial pressure. Thus, assessment of all potential causes of PH in a patient needs to consider PH associated with increased pulmonary vascular resistance, as well as with increased pulmonary blood flow [16].

The ratio of acceleration time over right ventricular ejection time in the main pulmonary artery is a relevant ultrasound Doppler index to assess precapillary pulmonary vascular resistance,[17] and relative shortening of pulmonary artery acceleration time has been associated with CDH severity [18]. In our patient, however, this index was not relevant, as right lung perfusion was supplied by the left ventricle, whereas left lung perfusion was supplied by the right ventricle. Persistence of a right-to-left flow through a tubular ductus arteriosus, which generally indicates increased right-sided cardiac pressures, also gave the opportunity, in this hemodynamic situation, to distribute part of the systemic volume flow and to reduce PH severity of the left lung, as exceptionally observed in adults [19].

Repeated echocardiography examinations performed in this patient found no evidence for post-capillary PH. Careful examination confirmed the absence of pulmonary vein stenosis, mitral stenosis, left ventricle dysfunction, left outflow tract obstacle, or aortic coarctation.

The unexpected occurrence of an increased pulmonary blood flow in the present CDH infant, led to the search for left-to-right shunt, and, ultimately, to the identification of an anomalous origin of the right pulmonary artery from the ascending aorta. The challenge was then to totally reverse the therapeutic strategy. Our objective was first to reduce pulmonary blood flow by increasing pulmonary vascular resistance, which involved suspension of pulmonary vasodilators, elevation of ventilation pressures, and permissive hypercapnia. In addition, we aimed to reduce systemic-to-pulmonary difference in vascular resistance. Therefore, we reduced norepinephrine dose, as this drug increases systemic vascular resistance but reduces pulmonary vascular resistance in experimental CDH [16].

In conclusion, this case reports the importance of going back to the basics of physiology in complex hemodynamic situations, especially when the expected evolution is not observed. Indeed, we usually only find what we are looking for.

## Data Availability

The dataset supporting the conclusions of this article is contained within the manuscript.

## Abbreviations

CDH: congenital diaphragmatic hernia
ECMO: extracorporeal membrane oxygenation
FiO_2_: fraction of inspired oxygen
MRI: magnetic resonance imaging
NICU: neonatal intensive care unit
iNO: inhaled nitric oxide
PCO_2_: partial pressure of carbon dioxide
PH: pulmonary hypertension
SpO_2_: pulse oximetry oxygen saturation
